# Potential Benefits of Ultra-High Field MRI for Embryonic and Fetal Brain Investigation: A Comprehensive Review

**DOI:** 10.3390/diagnostics16071026

**Published:** 2026-03-29

**Authors:** Dan Boitor, Mihaela Oancea, Alexandru Farcasanu, Simion Simon, Daniel Muresan, Ioana Cristina Rotar, Georgiana Irina Nemeti, Iulian Goidescu, Adelina Staicu, Mihai Surcel

**Affiliations:** 1 Department of Obstetrics and Gynecology, “Iuliu Hatieganu” University of Medicine and Pharmacy, 40012 Cluj-Napoca, Romania; dan.boitor@umfcluj.ro (D.B.); daniel.muresan@umfcluj.ro (D.M.); cristina.rotar@umfcluj.ro (I.C.R.); georgiana.nemeti@umfcluj.ro (G.I.N.); iulian.goidescu@umfcluj.ro (I.G.); adelina.staicu@umfcluj.ro (A.S.); mihai.surcel@umfcluj.ro (M.S.); 2 INSPIRE Platform, Babes-Bolyai University, 40028 Cluj-Napoca, Romania; stefan.farcasanu@gmail.com (A.F.); simion49nmr@gmail.com (S.S.); 3 Amethyst Radiotherapy Center Cluj, 407280 Florești, Romania

**Keywords:** ultra-high-field MRI, embryonic brain, fetal brain, brain development

## Abstract

Ultra-high-field (UHF) magnetic resonance imaging, defined as imaging at field strengths of 7 Tesla (7T) and above, represents a frontier technology in neuroimaging with emerging applications in prenatal brain research. This narrative review examines the current evidence on the potential benefits of UHF-MRI for investigating embryonic and fetal brain development. Through analysis of 97 studies identified across multiple databases, we find that UHF-MRI offers substantial advantages in spatial resolution, tissue contrast, and anatomical detail compared to conventional clinical field strengths (1.5T and 3T). The primary applications to date have been in ex vivo imaging of post-mortem fetal specimens and preclinical animal models, where UHF-MRI has enabled unprecedented visualization of laminar cortical organization, early sulcation patterns, microstructural development, and subtle anatomical features critical for understanding normal and abnormal neurodevelopment. Key benefits include enhanced delineation of transient developmental zones, improved characterization of cortical folding, superior detection of subtle malformations, and the ability to create high-resolution three-dimensional atlases of fetal brain development. However, significant technical and safety challenges currently limit in utero human applications, including concerns about specific absorption rate, acoustic noise, and fetal motion artifacts. This review identifies critical knowledge gaps and future directions for translating UHF-MRI technology to clinical prenatal diagnostics.

## 1. Introduction

The human brain undergoes remarkable transformation during prenatal development, with critical processes including neurogenesis, neuronal migration, cortical lamination, and the emergence of sulcation and gyrification occurring in a tightly orchestrated temporal sequence [1,2]. Understanding these developmental processes is essential for identifying abnormalities, predicting neurodevelopmental outcomes, and advancing our knowledge of both typical and atypical brain development [3,4]. Magnetic resonance imaging (MRI) has emerged as a non-invasive tool for visualizing fetal brain structure in vivo, complementing ultrasound with superior soft tissue contrast and the ability to image the entire brain regardless of fetal position or maternal body habitus [5,6].

Conventional fetal MRI is typically performed at clinical field strengths of 1.5 Tesla (1.5T) or 3.0 Tesla (3T), which provide adequate visualization of major brain structures and gross anatomical abnormalities [7,8]. However, the spatial resolution and tissue contrast achievable at these field strengths are often insufficient to visualize fine anatomical details, transient developmental zones, and subtle microstructural features that are critical for understanding early brain development [9,10]. Ultra-high-field (UHF) MRI, defined as imaging at 7T and above, offers theoretical advantages in signal-to-noise ratio (SNR) and contrast-to-noise ratio (CNR) that scale approximately linearly with field strength, potentially enabling visualization of anatomical structures and developmental processes that are invisible at lower field strengths [11,12].

Despite these theoretical advantages, the application of UHF-MRI to fetal brain imaging remains in its early stages, with most studies conducted ex vivo on post-mortem specimens or in preclinical animal models [1,13]. This narrative review aims to map the current evidence on the potential benefits of UHF-MRI for embryonic and fetal brain investigation, identify key applications and findings, examine technical challenges, and outline future directions for this emerging field.

## 2. Background and Theoretical Foundations

### 2.1. Principles of Ultra-High-Field MRI

The fundamental advantage of UHF-MRI stems from the relationship between magnetic field strength and signal intensity. The signal-to-noise ratio in MRI increases approximately linearly with field strength, meaning that a 7T scanner theoretically provides more than twice the SNR of a 3T scanner and nearly five times that of a 1.5T scanner [11]. This increased SNR can be leveraged in three primary ways: (1) to achieve higher spatial resolution while maintaining adequate image quality, (2) to reduce acquisition time while maintaining resolution, or (3) to improve contrast-to-noise ratio for better tissue differentiation [14].

At ultra-high field strengths, several tissue contrast mechanisms are enhanced. T1 relaxation times increase with field strength, providing improved gray-white matter contrast in T1-weighted images [15]. Susceptibility effects are amplified, enhancing contrast in T2-weighted and susceptibility-weighted imaging sequences [16]. These properties make UHF-MRI particularly well-suited for visualizing subtle anatomical boundaries and microstructural features in the developing brain [1,17].

### 2.2. Fetal Brain Development: Critical Periods and Structures

Human fetal brain development follows a highly orchestrated sequence of events spanning from early embryonic stages through birth and beyond [18,19]. During the first and second trimesters, key developmental milestones include the formation of the neural tube, emergence of primary brain vesicles, neuronal proliferation in germinal zones, and the beginning of neuronal migration [20]. The transient zones of the developing cerebral wall—including the ventricular zone, subventricular zone, intermediate zone, subplate, cortical plate, and marginal zone—exhibit distinct cellular compositions and signal characteristics on MRI [21,22].

Cortical folding, or gyrification, begins in the second trimester and continues through the third trimester and early postnatal period [23,24]. The emergence of primary sulci follows a predictable temporal and spatial pattern, with the Sylvian fissure appearing first around 14 weeks gestational age, followed by the calcarine, parieto-occipital, and cingulate sulci [1,25]. Visualization of these early sulcation patterns and the transient laminar organization of the fetal cortex require high spatial resolution and excellent tissue contrast, making this an ideal application for UHF-MRI [1,7].

### 2.3. Current State of Fetal MRI

Clinical fetal MRI is routinely performed at 1.5T and increasingly at 3T, using fast imaging sequences designed to minimize motion artifacts from fetal and maternal movement [26,27]. Standard protocols include T2-weighted single-shot fast spin echo sequences, which provide excellent anatomical detail of major brain structures, and increasingly, advanced techniques such as diffusion-weighted imaging, diffusion tensor imaging, and functional MRI [28]. While these techniques have revolutionized prenatal diagnosis and our understanding of fetal brain development, they remain limited in their ability to visualize fine anatomical details and subtle developmental abnormalities [29,30].

The transition to 3T fetal MRI has demonstrated improved image quality and diagnostic confidence compared to 1.5T, particularly for small structures and subtle abnormalities [31]. However, 3T imaging also introduces challenges including increased specific absorption rate (SAR), greater susceptibility to motion artifacts, and more pronounced *B*_0_ and *B*_1_ field inhomogeneities [32]. These challenges are further amplified at ultra-high field strengths, necessitating careful consideration of safety, technical feasibility, and clinical utility [33].

## 3. Methods and Scope

### 3.1. Search Strategy and Data Sources

This review was conducted following established methodological frameworks for narrative reviews. A comprehensive literature search was performed across multiple databases including Google Scholar and PubMed. Search queries were designed to capture studies investigating ultra-high-field MRI (7T, 9T, and higher) applications in embryonic and fetal brain imaging, prenatal neurodevelopment, and related preclinical models. The search strategy combined terms related to ultra-high-field MRI technology with terms describing fetal, embryonic, and prenatal brain imaging applications.

### 3.2. Inclusion Criteria and Study Selection

Studies were included if they: (1) utilized MRI at field strengths of 7T or higher, (2) focused on embryonic, fetal, or early developmental brain imaging, (3) reported on imaging techniques, anatomical findings, or developmental insights, or (4) provided relevant technical or methodological information applicable to prenatal brain imaging. Both human and animal model studies were included to capture the full scope of UHF-MRI applications in developmental neuroscience. Review articles and methodological papers were included to provide context and identify technical considerations.

### 3.3. Data Extraction and Analysis

We selected 30 relevant papers that were analyzed in detail, with systematic extraction of information on field strength, imaging modalities, study design, sample characteristics, key findings, and reported benefits of UHF-MRI. These articles were then manually reviewed to exclude those that were mistakenly selected or focused on unrelated methodologies, where MRI was only used as a supporting method without significant relevance to our review. This analysis forms the evidence base for the synthesis presented in subsequent sections.

## 4. Applications of Ultra-High-Field MRI in Fetal Brain Research

### 4.1. Ex Vivo Imaging of Human Fetal Specimens

The most established application of UHF-MRI in fetal brain research is ex vivo imaging of post-mortem human fetal specimens. Zhang et al. conducted a landmark study using 7.0T MRI to map fetal brain development in the second trimester, scanning 69 fetal specimens ranging from 12 to 22 gestational weeks (GW) [1]. This study demonstrated that 7T MRI could distinctly visualize developing structures including most sulci (except postcentral and intraparietal sulci), laminar organization described as layers with different signal intensities, and clearly visible basal nuclei. The laminar organization became typical after 16 weeks of gestational age, and the study enabled creation of three-dimensional visualization models that aided greatly in precise cognition of the immature brain [1]. Boitor-Borza et al. described the lamination of the ventral wall (subpallium) of telencephalic vesicles in a 9 GW embryo with 21 mm crown-rump length (CRL) [33]; they also depicted the ganglionic eminences by using micro-MRI at 7.04T in a series of six subjects ranging from 21 mm CRL (9 GW) to 85 mm CRL (14 GW).

High-resolution ex vivo MRI at ultra-high field strengths has proven particularly valuable for characterizing the detailed layer structures of the early human fetal brain. Wang et al. utilized high-field MRI to reveal detailed layer structures in early human fetal stages with histologic correlation, demonstrating that UHF-MRI can visualize transient developmental zones that are critical for understanding normal and abnormal cortical development [7]. In consideration of its historical relevance, we mention that Sbarbati et al. employed high-field MRI to study human brain development from the 10th to 16th week of gestational age, providing insights into the earliest stages of cerebral cortex formation [8].

The ability to create high-resolution three-dimensional atlases of fetal brain development represents a major contribution of UHF ex vivo imaging. Recently, authors demonstrated brain structures in a human embryo imaged with MR microscopy, showcasing the potential for UHF-MRI to visualize even earlier developmental stages with unprecedented detail [10,33]. These atlases serve as critical reference standards for interpreting in vivo fetal MRI and for identifying subtle developmental abnormalities [1,7].

### 4.2. Preclinical Animal Models

Ultra-high-field MRI has been extensively applied to preclinical animal models of fetal brain development, providing controlled experimental conditions and the ability to correlate imaging findings with histological and molecular analyses. Sawada et al. characterized fetal sulcation and gyrification in common marmosets using 7T ex vivo MRI, demonstrating that high-resolution imaging could quantify cortical folding patterns and correlate them with cortical volume and surface area throughout fetal development [2]. This study revealed that the outer subventricular zone in non-sulcal regions was thicker than in presumptive sulcal regions, preceding sulcal infolding—an observation that would be difficult to make without the high spatial resolution afforded by 7T MRI [2].

In rodent models, UHF-MRI has enabled in utero imaging of embryonic brain development and injury. Wu et al. performed in utero localized diffusion MRI of the embryonic mouse brain microstructure and injury, demonstrating the feasibility of longitudinal imaging studies to track developmental processes and responses to injury in living embryos [3]. Nakano et al. conducted longitudinal evaluation using preclinical 7T MRI and spectroscopy on prenatally alcohol-exposed rats, showing that UHF-MRI can detect subtle metabolic and structural changes associated with prenatal exposures [4].

The use of animal models at ultra-high-field strengths has been particularly valuable for validating imaging biomarkers and understanding the biological basis of MRI signal changes during development. These studies provide proof-of-concept for imaging techniques and analytical approaches that may eventually be translated to human fetal imaging [2,3,4].

### 4.3. Advanced Imaging Techniques at Ultra-High-Field

Beyond conventional anatomical imaging, UHF-MRI enables advanced techniques that provide complementary information about fetal brain development. Diffusion MRI at ultra-high field offers improved angular resolution and sensitivity to microstructural features, enabling more detailed characterization of white matter development and neuronal migration patterns [12]. Christiaens et al. reviewed the challenges and advances in in utero diffusion MRI, noting that higher field strengths could potentially overcome some of the SNR limitations that currently constrain diffusion imaging in the fetus [12].

Magnetic resonance spectroscopy (MRS) benefits substantially from increased field strength, with improved spectral resolution and sensitivity enabling detection of a broader range of metabolites and more subtle metabolic changes [4]. Nakano et al. demonstrated the application of 7T MRS to detect metabolic alterations in prenatally exposed rat brains, suggesting potential applications for assessing fetal brain metabolism and detecting metabolic disorders [4].

Functional MRI (fMRI) and connectivity studies may also benefit from ultra-high field strengths, though these applications remain largely unexplored in the fetal context due to technical challenges. The improved SNR and BOLD contrast at 7T could potentially enable detection of subtle functional activity patterns and connectivity changes during fetal brain development [5].

## 5. Key Benefits and Comparative Analysis

### 5.1. Enhanced Spatial Resolution

The most consistently reported benefit of UHF-MRI for fetal brain imaging is dramatically improved spatial resolution. Zhang et al. achieved visualization of laminar cortical organization and individual sulci in second-trimester fetuses using 7T MRI, with detail that would be unattainable at clinical field strengths [1]. This enhanced resolution enables identification of subtle anatomical features and developmental landmarks that are critical for accurate staging of brain development and detection of abnormalities [7,10].

The ability to visualize transient developmental zones—including the ventricular zone, subventricular zone, intermediate zone, subplate, and cortical plate—represents a particular advantage of UHF-MRI. Wang et al. demonstrated that high-field MRI could reveal detailed layer structures with histologic correlation, providing a non-invasive window into the cellular architecture of the developing brain [7]. This capability has important implications for understanding disorders of neuronal migration and cortical malformations [21].

### 5.2. Superior Tissue Contrast and Anatomical Detail

Ultra-high field MRI provides superior tissue contrast compared to lower field strengths, enabling better differentiation of gray and white matter, visualization of subtle anatomical boundaries, and detection of small structures. Zhang et al. reported that laminar organization could be delineated as layers with different signal intensities on 7T MRI, with basal nuclei distinctly visible even in early second-trimester specimens [1]. This enhanced contrast is particularly valuable for characterizing the complex, heterogeneous tissue architecture of the developing brain [17].

Sbarbati et al. utilized high-field MRI to perform three-dimensional profiling of the cerebral cortex in human fetuses, demonstrating that UHF-MRI could capture the emergence of cortical folding patterns with unprecedented detail [21]. The ability to visualize early sulcation patterns and quantify cortical surface morphology has important applications for understanding the mechanisms of gyrification and identifying abnormal folding patterns associated with developmental disorders [2,23].

### 5.3. Quantitative Assessment of Brain Development

UHF-MRI enables quantitative assessment of fetal brain development with greater precision than conventional imaging. Zhang et al. used 7T MRI to quantify growth rates of different brain structures, demonstrating linear increases with gestational age [1]. Sawada et al. quantified gyrification index and sulcation index in fetal marmosets, showing close correlations with cortical volume and surface area [2]. These quantitative metrics provide objective measures of brain development that can be used to establish normative trajectories and identify deviations from typical development [19,20].

The improved SNR at ultra-high field also enables more sophisticated quantitative imaging techniques, including quantitative T1 and T2 mapping, diffusion tensor imaging with higher angular resolution, and metabolic imaging with MR spectroscopy [18]. Clouchoux et al. reviewed novel applications of quantitative MRI for the fetal brain, noting that higher field strengths could expand the range of quantitative biomarkers available for assessing fetal brain development [18].

### 5.4. Three-Dimensional Visualization and Atlas Construction

A major contribution of UHF ex vivo imaging has been the creation of high-resolution three-dimensional atlases of fetal brain development. Zhang et al. reconstructed three-dimensional visualization models from 7T MRI data, providing detailed anatomical references for different gestational ages [1]. These atlases serve multiple purposes: they provide normative references for interpreting clinical fetal MRI, enable automated segmentation and analysis of fetal brain structures, and facilitate education and research in developmental neuroscience [16,19].

The level of anatomical detail achievable with UHF-MRI enables visualization of structures and features that are not visible on conventional imaging. Kunieda et al. demonstrated brain structures in a human embryo using MR microscopy, revealing anatomical details comparable to histological sections but in three dimensions and without tissue destruction [10]. This capability is particularly valuable for rare specimens and for creating comprehensive developmental atlases spanning the full range of gestational ages [8].

### 5.5. Comparative Analysis: UHF-MRI vs. Clinical Field Strengths

While direct head-to-head comparisons of different field strengths for fetal brain imaging are limited, the available evidence suggests substantial advantages for UHF-MRI in research and ex vivo applications. Studies using 3T fetal MRI have demonstrated improved image quality compared to 1.5T, particularly for visualization of small structures and subtle abnormalities [31]. Welsh et al. reviewed fetal MRI at 3.0T, noting improvements in SNR and spatial resolution but also increased technical challenges [23]. 3-T MRI offers superior image quality and resolution for fetal imaging, particularly in neurological and body indications, but requires careful management of increased artifacts and adherence to specific protocols to ensure safety and diagnostic accuracy [34].

The transition from 3T to 7T represents an even larger increase in field strength, with corresponding improvements in SNR and resolution. However, this comes at the cost of increased technical complexity, longer acquisition times (for ex vivo imaging), and currently prohibitive safety concerns for in utero human imaging [33]. The optimal field strength for a given application depends on the specific research or clinical question, the required spatial and temporal resolution, and practical considerations of scanner availability and expertise [11,14].

## 6. Technical Considerations and Challenges

### 6.1. Safety Considerations for in Utero Imaging: Specific Absorption Rate, Acoustic Noise, Motion-Induced Electric Fields

The primary barrier to in utero human fetal imaging at ultra-high field strengths is safety. Specific absorption rate (SAR), which measures the rate of radiofrequency energy deposition in tissue, increases with the square of the field strength. At 7T, SAR can easily exceed regulatory limits, particularly for sequences with high flip angles or rapid repetition rates. While SAR can be managed through sequence optimization and careful monitoring, the lack of comprehensive safety data for fetal exposure to UHF-MRI has prevented clinical applications [32].

Acoustic noise is another significant concern, as the gradient switching required for MRI produces loud sounds that increase in intensity with field strength. While the fetus is partially protected by amniotic fluid and maternal tissues, the potential effects of prolonged exposure to intense acoustic noise during critical developmental periods remain uncertain [26]. Additional safety considerations include the effects of static magnetic field exposure on fetal development, though current evidence suggests that static fields up to 3T are safe for fetal imaging [27].

Movement in a spatially nonuniform static *B*_0_ converts to a time-varying magnetic flux in tissues, producing electric fields by Faraday induction. Induced electric fields and current densities scale roughly linearly with dB/dt and thus with *B*_0_ × velocity. Observed and modeled biological effects from motion-induced fields principally include sensory phenomena, potential peripheral nerve effects, and theoretical cardiac excitation risk. There is insufficient evidence in the supplied literature to provide measured values of typical fetal movement velocities or direct fetal-specific thresholds for motion-induced electrical stimulation.

Motion induction modeling implies larger induced electric fields for equivalent motion at 7T versus 1.5/3T because stray-field magnitudes and gradients increase with central field strength, so potential for motion-induced physiological effects is higher at UHF [35]. Nevertheless, no clinical cases of fetal harm attributable to motion-induced currents are reported in the supplied corpus.

Regulatory guidance from International Commission on Non-Ionizing Radiation Protection (ICNIRP) and Institute of Electrical and Electronics Engineers (IEEE) is used to derive practical limits (e.g., recommendations about limiting static *B*_0_ and controlling movement speed near high-field magnets). Nevertheless, exact numeric basic-restriction values for induced electric fields in fetal tissues are not provided in the reviewed papers.

### 6.2. Technical Challenges: Motion, Field Inhomogeneity, and Artifacts

In vivo fetal MRI is intrinsically challenged by unpredictable fetal and maternal motion that disrupts 3D encoding. Fetal motion represents a fundamental challenge for in utero MRI at any field strength, but the problem is exacerbated at ultra-high field due to longer acquisition times required for high-resolution imaging. While ex vivo imaging eliminates motion artifacts, in utero imaging would require fast imaging sequences and motion correction strategies specifically optimized for UHF-MRI [28]. Advanced techniques such as real-time imaging, retrospective motion correction, and slice-to-volume registration have been developed for clinical field strengths but need to be adapted for UHF applications [29,36].

Ultrahigh fields further introduce higher RF energy deposition (SAR), *B*_0_/*B*_1_ inhomogeneities and susceptibility artifacts that complicate sequence design and make it harder to realize nominal resolution gains in routine clinical fetal scanning [37]. *B*_0_ (static) and *B*_1_ (transmit/radiofrequency) field inhomogeneities in MRI cause image artifacts, signal loss, and quantification errors, particularly at higher field strengths like 3.0T. *B*_0_ and *B*_1_ field inhomogeneities increase with field strength, leading to signal intensity variations, geometric distortions, and artifacts that can degrade image quality [32]. The small size and complex geometry of the fetal brain, combined with the heterogeneous magnetic susceptibility of surrounding maternal tissues, create particularly challenging conditions for achieving uniform field distributions at 7T [14]. Specialized shimming techniques, parallel transmission, and post-processing corrections are required to mitigate these effects [15]. Nevertheless, there is insufficient evidence in the searched literature that 7T routinely provides better spatial resolution than optimized 3T for in vivo fetal imaging without advanced motion control, reconstruction, and UHF mitigation strategies.

### 6.3. Practical Considerations: Scanner Availability and Expertise

Ultra-high-field MRI scanners remain relatively rare, with most 7T systems located at major research institutions. The high cost of UHF-MRI systems, specialized infrastructure requirements (including enhanced radiofrequency shielding and cooling systems), and need for specialized technical expertise limit widespread availability [11]. For fetal brain imaging specifically, the development of specialized radiofrequency coils, imaging protocols, and analysis pipelines requires substantial investment and interdisciplinary collaboration [13].

The long acquisition times typical of high-resolution UHF-MRI (often hours for ex vivo imaging) are practical for post-mortem specimens but would be prohibitive for in utero imaging even if safety concerns were addressed [1,7]. Balancing the desire for maximum resolution with practical constraints on scan time represents an ongoing challenge in UHF-MRI protocol development [14].

## 7. Discussion

### 7.1. Current State of the Field

This narrative review reveals that UHF-MRI has made significant contributions to our understanding of fetal brain development, primarily through ex vivo imaging of post-mortem human specimens and preclinical animal models. The enhanced spatial resolution and tissue contrast afforded by 7T and higher field strengths have enabled visualization of anatomical details and developmental processes that were previously accessible only through histological examination [1,7,10]. These advances have led to the creation of high-resolution developmental atlases, improved understanding of cortical lamination and folding, and new insights into the timing and spatial patterns of brain development [2,8,21].

However, the application of UHF-MRI to in utero human fetal imaging remains largely aspirational. Safety concerns, technical challenges, and practical limitations have prevented clinical translation of this technology for prenatal diagnosis [33]. The current evidence base consists primarily of ex vivo studies and animal models, with limited direct evidence regarding the clinical utility of UHF-MRI for prenatal diagnosis or prognosis [1,2,3,4].

### 7.2. Strengths and Limitations of Current Evidence

The primary strength of the current evidence is the consistent demonstration that UHF-MRI can visualize fetal brain structures with unprecedented detail in controlled ex vivo settings. Multiple studies across different research groups and using different field strengths (7T, 9T, 11.7T) have confirmed the technical feasibility and scientific value of UHF-MRI for developmental neuroscience [1,2,7,8,10]. The correlation of imaging findings with histological analysis provides validation of the anatomical accuracy of UHF-MRI [7].

However, several important limitations must be acknowledged. First, the evidence base is dominated by ex vivo studies, which eliminate motion artifacts but may not fully represent in vivo tissue properties due to fixation effects and the absence of blood flow [17]. Second, most studies involve small sample sizes and focus on specific gestational age ranges, limiting the generalizability of findings [1,2]. Third, there is a lack of standardization in imaging protocols, analysis methods, and reporting of results, making it difficult to compare findings across studies [13].

The translation gap between ex vivo research applications and potential clinical in utero imaging represents a critical limitation. While ex vivo UHF-MRI has clear value for research, atlas construction, and validation of imaging biomarkers, the clinical impact depends on the ability to safely and practically image living fetuses [33]. Current evidence provides limited guidance on how to bridge this gap [26,27].

### 7.3. Implications for Prenatal Diagnosis and Neurodevelopmental Research

Despite current limitations, UHF-MRI has important implications for both prenatal diagnosis and basic neurodevelopmental research. The high-resolution atlases created through ex vivo UHF-MRI serve as critical reference standards for interpreting clinical fetal MRI at lower field strengths [1,19]. Understanding the detailed anatomy and developmental trajectories revealed by UHF-MRI can improve diagnostic accuracy and confidence when evaluating fetuses with suspected brain abnormalities [9,11].

For neurodevelopmental research, UHF-MRI provides a powerful tool for investigating the mechanisms of brain development, the effects of genetic and environmental factors on prenatal brain structure, and the origins of neurodevelopmental disorders [3,4]. The ability to visualize transient developmental zones, quantify cortical folding patterns, and assess microstructural development with high precision enables research questions that were previously unanswerable [2,7,21]. Ultra-high-field MRI provides substantially higher signal-to-noise ratio that can be traded for improved spatial resolution and contrast, making it attractive for resolving small anatomical features [38]. Where motion is absent or well controlled, studies show clearer depiction of fine neonatal anatomy at 7T and excellent soft-tissue contrast in postmortem fetal tissues, indicating the modality’s potential to outperform 3T under ideal conditions [39,40].

Preclinical applications of UHF-MRI in animal models have significant value for translational research, enabling longitudinal studies of brain development, experimental manipulations, and validation of imaging biomarkers under controlled conditions [2,3,4]. These studies provide proof-of-concept for imaging techniques and analytical approaches that may eventually be adapted for human applications [13].

### 7.4. Clinical Results Using UHF MRI

Studies comparing 7T versus conventional (1.5T/3T) MRI in cohorts with epilepsy or cortical malformations report measurable diagnostic gains and variable surgical impact.

Across cohorts the principal added value of 7T was consistent increased detection and better delineation of subtle malformations of cortical development relevant to epilepsy, with patterns and sequences repeatedly implicated. Detection rates were improved at UHF MRI, with 6/21 (29%) new lesions at 7T in a 21-patient cohort [41], and 9/40 (23%) additional lesions in a 40-patient presurgical cohort [42]. Lesion types benefiting most are focal cortical dysplasia (particularly FCD IIb), periventricular nodular heterotopia, and polymicrogyria, which were repeatedly reported as better visualized or more sharply delineated at 7T [42].

Concerning the clinical impact on management, the reviewed cohorts provide examples where 7T findings affected invasive evaluation or surgical decisions and where resection of 7T-identified lesions correlated with postoperative outcomes. Surgical conversion from non-lesional to lesional resulted in patients who were earlier deemed MRI-negative at 1.5/3T being reclassified as lesional after undergoing 7T imaging and subsequently moving forward to surgery with histopathologic validation of focal cortical dysplasia (FCD) or malformations of cortical development (MCD) [41,42].

### 7.5. Gaps in Current Knowledge

Several critical gaps in current knowledge limit our understanding of the potential benefits and limitations of UHF-MRI for fetal brain imaging. First, there is insufficient evidence regarding the safety of in utero UHF-MRI exposure, particularly regarding potential effects on neurodevelopment, hearing, and other organ systems. Comprehensive safety studies in appropriate animal models are needed before human in utero imaging can be considered [26].

Second, the technical feasibility of in utero UHF-MRI remains uncertain. While motion correction and fast imaging techniques have been developed for clinical field strengths, their performance at 7T and above is largely unknown [28]. Research is needed to develop and validate UHF-MRI protocols specifically optimized for in utero fetal brain imaging [29].

Third, the clinical utility of UHF-MRI for prenatal diagnosis has not been established. Even if technical and safety challenges can be overcome, it remains unclear whether the additional anatomical detail provided by UHF-MRI would meaningfully improve diagnostic accuracy, clinical decision-making, or patient outcomes compared to optimized 3T imaging [31]. Comparative effectiveness studies would be needed to justify the substantial costs and complexity of UHF-MRI for clinical applications [11].

Finally, there is limited understanding of how to optimally leverage the unique capabilities of UHF-MRI for specific research and clinical questions. What are the most valuable applications of UHF-MRI in fetal brain research? Which anatomical structures or developmental processes benefit most from ultra-high-field imaging? How should UHF-MRI be integrated with other imaging modalities and assessment techniques? These questions require systematic investigation [13,14].

## 8. Conclusions

Ultra-high-field MRI at 7T and above offers substantial potential benefits for investigating embryonic and fetal brain development, including dramatically improved spatial resolution, superior tissue contrast, enhanced visualization of transient developmental zones, and the ability to create high-resolution three-dimensional atlases. Current applications have been primarily in ex vivo imaging of post-mortem human specimens and preclinical animal models, where UHF-MRI has provided unprecedented insights into cortical lamination, sulcation patterns, and microstructural development [1,2,7,10].

However, significant technical and safety challenges currently prevent in utero human applications of UHF-MRI. Concerns about specific absorption rate, acoustic noise exposure, motion artifacts, and field inhomogeneities must be addressed before clinical translation can be considered [26,27,32]. Even if these challenges can be overcome, the clinical utility of UHF-MRI for prenatal diagnosis remains to be established through rigorous comparative effectiveness studies [31]. Ultra-high-field MRI (≥7T) has the physics to deliver higher SNR and finer spatial detail, but unpredictable fetal and maternal motion plus ultrahigh-field specific artifacts and safety constraints commonly prevent routinely achieving better in vivo fetal resolution than optimized 3T protocols.

In the near term, the most valuable contributions of UHF-MRI to fetal brain research will likely come from continued advancement of ex vivo imaging applications, creation of comprehensive developmental atlases, and preclinical studies in animal models [1,13]. These applications provide important scientific insights and may indirectly improve clinical care through better understanding of brain development and validation of imaging biomarkers applicable at clinical field strengths [11,19].

Looking forward, sustained interdisciplinary collaboration, technical innovation, and careful attention to safety will be required to realize the full potential of UHF-MRI for understanding and monitoring fetal brain development. While the path to clinical translation remains uncertain, the scientific value of UHF-MRI for developmental neuroscience is already well established [1,2,7,10]. Continued investment in this technology and its applications promises to yield important insights into one of the most remarkable processes in human biology, the development of the brain.

## 9. Future Directions and Recommendations

### 9.1. Advancing Ex Vivo Applications

In the near term, the most promising applications of UHF-MRI for fetal brain research lie in continued advancement of ex vivo imaging. Priorities include (1) expanding high-resolution atlases to cover the full range of gestational ages from early embryonic stages through term, (2) developing standardized imaging protocols and analysis pipelines to enable multi-center studies and data sharing, (3) integrating UHF-MRI with complementary techniques such as histology, immunohistochemistry, and molecular analysis to provide comprehensive characterization of brain development, and (4) applying UHF-MRI to study abnormal brain development in fetuses with genetic disorders, structural malformations, or exposure to teratogens [1,7,10].

The creation of publicly available, high-resolution fetal brain atlases based on UHF-MRI would be a valuable resource for the research community. Such atlases could serve as reference standards for automated segmentation algorithms, provide normative data for quantitative analysis of clinical fetal MRI, and facilitate education in developmental neuroanatomy [16,19]. Collaborative efforts to pool data across institutions and create comprehensive atlases should be prioritized [13].

### 9.2. Preclinical Research and Translational Studies

Preclinical animal models will continue to play a critical role in advancing UHF-MRI for fetal brain imaging. Key priorities include (1) conducting comprehensive safety studies of in utero UHF-MRI exposure in appropriate animal models, with assessment of neurodevelopmental, auditory, and other outcomes, (2) developing and validating motion-robust imaging sequences and reconstruction algorithms for in utero imaging at ultra-high field, (3) establishing imaging biomarkers of brain development and injury that can be translated to human applications, and (4) investigating the biological basis of MRI signal changes during development through correlation with histological and molecular analyses [2,3,4].

Longitudinal studies in animal models, tracking brain development from embryonic stages through postnatal maturation, would provide valuable insights into developmental trajectories and the effects of genetic and environmental factors [4]. The ability to perform controlled experiments and obtain tissue for validation studies makes animal models an essential complement to human ex vivo imaging [2].

### 9.3. Technical Development for in Utero Imaging

Substantial technical development is required before in utero human fetal imaging at ultra-high field can be considered feasible.

Building on previous 7T micro-MRI investigations of early human fetal brain development [33], future research directions will leverage the ultra-high-field 11.7T MRI infrastructure available within the INSPIRE research platform, realized based on the INSPIRE-I (SMIS 2014+ 127725) and INSPIRE-II (SMIS 2021+ 324771) research projects, to further enhance spatial resolution and microstructural characterization.

Priorities include (1) development of specialized radiofrequency coils optimized for fetal imaging at 7T, with attention to SAR management and field homogeneity, (2) optimization of fast imaging sequences that minimize SAR while maintaining adequate SNR and resolution, (3) development and validation of motion correction strategies specifically designed for UHF fetal imaging, (4) investigation of parallel imaging and compressed sensing techniques to accelerate acquisition and reduce SAR, and (5) development of real-time monitoring systems to ensure fetal safety during UHF-MRI [28,29,32].

Collaboration between MRI physicists, biomedical engineers, and fetal medicine specialists will be essential for addressing these technical challenges. Phantom studies and simulations can provide initial data on field distributions, SAR, and image quality before proceeding to in vivo studies [14,15].

### 9.4. Clinical Translation and Validation

If technical and safety challenges can be adequately addressed, clinical translation of UHF-MRI for fetal brain imaging will require rigorous validation studies. Key steps include (1) pilot studies in pregnant women at low risk, with careful monitoring of maternal and fetal safety, (2) comparison of diagnostic accuracy and image quality between UHF-MRI and optimized 3T imaging for specific clinical indications, (3) assessment of the added value of UHF-MRI for clinical decision-making and patient outcomes, (4) cost-effectiveness analysis to determine whether the benefits of UHF-MRI justify the substantial additional costs, and (5) development of clinical guidelines for appropriate use of UHF-MRI in prenatal diagnosis [31].

It is important to note that clinical translation may not be appropriate or necessary for all applications. UHF-MRI may remain primarily a research tool, with its main clinical impact being indirect—through improved understanding of brain development, creation of reference atlases, and validation of imaging biomarkers that can be applied at clinical field strengths [11,13]. Realizing UHF advantages in vivo will require dedicated motion-robust acquisitions and reconstruction pipelines plus safety/coil solutions tailored to the pregnant abdomen [36].

### 9.5. Interdisciplinary Collaboration and Data Sharing

Advancing UHF-MRI for fetal brain research will require sustained interdisciplinary collaboration among MRI physicists, biomedical engineers, developmental neurobiologists, fetal medicine specialists, neonatologists, and other stakeholders. Establishing collaborative networks, shared resources, and standardized protocols will be essential for making progress on the complex technical, safety, and clinical questions that must be addressed [13].

Data sharing and open science practices should be prioritized to maximize the impact of UHF-MRI research. Given the rarity of UHF-MRI scanners and the challenges of acquiring fetal brain imaging data, sharing of raw data, processed images, and analysis tools would accelerate progress and enable research questions that require large sample sizes. Development of data standards, repositories, and sharing agreements should be pursued [19].

## Data Availability

Data sharing is not applicable because no new data were created.

## Abbreviations

The following abbreviations are used in this manuscript:

MRI	magnetic resonance imaging
UHF	ultra-high field
SNR	signal-to-noise ratio
CNR	contrast-to-noise ratio
SAR	specific absorption rate
MRS	magnetic resonance spectroscopy
fMRI	functional MRI
BOLD	Blood Oxygen Level-Dependent

## References

[1] Karimi D., Calixto C., Snoussi H., Li B., Cortes-Albornoz M.C., Velasco-Annis C., Rollins C., Pierotich L., Jaimes C., Gholipour A. (2026). Detailed Delineation of the Fetal Brain in Diffusion MRI via Multi-Task Learning. IEEE Trans. Med. Imaging.

[2] Sawada K., Hikishima K., Murayama A.Y., Okano H.J., Sasaki E., Okano H. (2014). Fetal sulcation and gyrification in common marmosets (*Callithrix jacchus*) obtained by ex vivo magnetic resonance imaging. Neuroscience.

[3] Wu D., Lei J., Rosenzweig J.M., Burd I., Zhang J. (2015). In utero localized diffusion MRI of the embryonic mouse brain microstructure and injury. J. Magn. Reson. Imaging.

[4] Nakano T., Natsuyama T., Tsuji N., Katayama N., Ueda J., Saito S. (2023). Longitudinal Evaluation Using Preclinical 7T-Magnetic Resonance Imaging/Spectroscopy on Prenatally Dose-Dependent Alcohol-Exposed Rats. Metabolites.

[5] Yetisir F., Turk E.A., Feldman H.A., Gagoski B., Didier R.A., Barnewolt C., Estroff J.A., Wald L.L., Adalsteinsson E., Grant P.E. (2025). Fetal MRI: Radiofrequency Safety Assessment at 3 Tesla. J. Magn. Reson. Imaging.

[6] Jiang S., Xue H., Glover A., Rutherford M., Rueckert D., Hajnal J.V. (2007). MRI of moving subjects using multislice snapshot images with volume reconstruction (SVR): Application to fetal, neonatal, and adult brain studies. IEEE Trans. Med. Imaging.

[7] Wang R., Dai G., Takahashi E. (2015). High Resolution MRI Reveals Detailed Layer Structures in Early Human Fetal Stages: In Vitro Study with Histologic Correlation. Front. Neuroanat..

[8] Sbarbati A., Marzola P., Simonati A., Nicolato E., Osculati F. (1998). High-field magnetic resonance imaging of the developing human brain from the 10th to the 16th week of gestational Age. Acta Anat..

[9] Saleem S.N. (2013). Fetal magnetic resonance imaging (MRI): A tool for a better understanding of normal and abnormal brain development. J. Child. Neurol..

[10] Kunieda K., Makihara K., Yamada S., Yamaguchi M., Nakamura T., Terada Y. (2025). Brain Structures in a Human Embryo Imaged with MR Microscopy. Magn. Reson. Med. Sci..

[11] Tarui T., Gimovsky A.C., Madan N. (2024). Fetal neuroimaging applications for diagnosis and counseling of brain anomalies: Current practice and future diagnostic strategies. Semin. Fetal Neonatal Med..

[12] Christiaens D., Slator P.J., Cordero-Grande L., Price A.N., Deprez M., Alexander D.C., Rutherford M., Hajnal J.V., Hutter J. (2019). In Utero Diffusion MRI: Challenges, Advances, and Applications. Top. Magn. Reson. Imaging.

[13] Rutherford M., Jiang S., Allsop J., Perkins L., Srinivasan L., Hayat T., Kumar S., Hajnal J. (2008). MR imaging methods for assessing fetal brain development. Dev. Neurobiol..

[14] Counsell S.J., Arichi T., Arulkumaran S., Rutherford M.A. (2019). Fetal and neonatal neuroimaging. Handbook of Clinical Neurology.

[15] Im K., Huang H., Roberts T.P.L. (2021). Advanced fetal MRI. Advances in Magnetic Resonance Technology and Applications.

[16] Zhang J., Chen R., Tian C., Zhu K., Ren G., Bao A., Shen Y., Li X., Zhang Y., Qiu W. (2025). Ex vivo Magnetic Resonance Imaging of the Human Fetal Brain. Dev. Neurosci..

[17] Vasung L., Charvet C.J., Shiohama T., Gagoski B., Levman J., Takahashi E. (2019). Ex vivo fetal brain MRI: Recent advances, challenges, and future directions. Neuroimage.

[18] Clouchoux C., Limperopoulos C. (2012). Novel applications of quantitative MRI for the fetal brain. Pediatr. Radiol..

[19] Studholme C. (2011). Mapping fetal brain development in utero using magnetic resonance imaging: The Big Bang of brain mapping. Annu. Rev. Biomed. Eng..

[20] Benkarim O.M., Sanroma G., Zimmer V.A., Muñoz-Moreno E., Hahner N., Eixarch E., Camara O., González Ballester M.A., Piella G. (2017). Toward the automatic quantification of in utero brain development in 3D structural MRI: A review. Hum. Brain Mapp..

[21] Sbarbati A., Pizzini F., Fabene P.F., Nicolato E., Marzola P., Calderan L., Simonati A., Longo L., Osculati A., Beltramello A. (2004). Cerebral cortex three-dimensional profiling in human fetuses by magnetic resonance imaging. J. Anat..

[22] Glenn O.A., Barkovich J. (2006). Magnetic resonance imaging of the fetal brain and spine: An increasingly important tool in prenatal diagnosis: Part 2. AJNR Am. J. Neuroradiol..

[23] Welsh R.C., Nemec U., Thomason M.E. (2011). Fetal magnetic resonance imaging at 3.0 T. Top. Magn. Reson. Imaging.

[24] Rousseau F., Studholme C., Jardri R., Thomason M., Reissland N., Kisilevsky B.S. (2016). In Vivo Human Fetal Brain Analysis Using MR Imaging. Medical Image Analysis of the Developing Brain.

[25] Schöpf V., Dittrich E., Berger-Kulemann V., Kasprian G., Kollndorfer K., Prayer D. (2013). Zukunftsweisende MRT-Techniken des fetalen Gehirns [Advanced MRI techniques of the fetal brain]. Radiologe.

[26] Grecco G.G., Mork B.E., Huang J.Y., Metzger C.E., Haggerty D.L., Reeves K.C., Gao Y., Hoffman H., Katner S.N., Masters A.R. (2021). Prenatal methadone exposure disrupts behavioral development and alters motor neuron intrinsic properties and local circuitry. eLife.

[27] Holman R., Wu W., Tang Q., Tsikouras P., Rath W., Von Tempelhoff G.F., Nikolettos N. (2022). Common Indications and Techniques in Prenatal MRI. Ectopic Pregnancy and Prenatal Diagnosis.

[28] Jakab A., Pogledic I., Schwartz E., Gruber G., Mitter C., Brugger P.C., Langs G., Schöpf V., Kasprian G., Prayer D. (2015). Fetal Cerebral Magnetic Resonance Imaging Beyond Morphology. Semin. Ultrasound CT MR.

[29] Lautarescu A., Bonthrone A.F., Bos B., Barratt B., Counsell S.J. (2024). Advances in fetal and neonatal neuroimaging and everyday exposures. Pediatr. Res..

[30] Rutherford M.A. (2009). Magnetic resonance imaging of the fetal brain. Curr. Opin. Obstet. Gynecol..

[31] Glenn O.A., Barkovich A.J. (2006). Magnetic resonance imaging of the fetal brain and spine: An increasingly important tool in prenatal diagnosis, part 1. AJNR Am. J. Neuroradiol..

[32] Sundgren P.C., Dong Q., Gómez-Hassan D., Mukherji S.K., Maly P., Welsh R. (2004). Diffusion tensor imaging of the brain: Review of clinical applications. Neuroradiology.

[33] Boitor-Borza D., Turcu F., Farcasanu S., Crivii C. (2021). Early development of human ganglionic eminences assessed in vitro by using 7.04 Tesla micro-MRI—A pilot study. Med. Pharm. Rep..

[34] Manganaro L., Capuani S., Gennarini M., Miceli V., Ninkova R., Balba I., Galea N., Cupertino A., Maiuro A., Ercolani G. (2023). Fetal MRI: What’s new? A short review. Eur. Radiol. Exp..

[35] Tang F., Giaccone L., Hao J., Freschi F., Wu T., Crozier S., Liu F. (2022). Exposure of Infants to Gradient Fields in a Baby MRI Scanner. Bioelectromagnetics.

[36] Bridgen P., Tomi-Tricot R., Uus A., Cromb D., Quirke M., Almalbis J., Bonse B., De la Fuente Botella M., Maggioni A., Cio P.D. (2024). High resolution and contrast 7 tesla MR brain imaging of the neonate. Front. Radiol..

[37] Fuente M.D.L., Ilie C., Bridgen P., Bonse B., Quirke M., Almabis J., Cromb D., Cawley P., Tomi-Tricot R., De Vita E. (2023). 823 Ultra-high field 7 Tesla Magnetic Resonance Brain Imaging in Neonates: Feasibility studies. Arch. Dis. Child..

[38] Mahmoud A., Tomi-Tricot R., Leitão D., Bridgen P., Price A.N., Uus A., Boutillon A., Lawrence A.J., Cromb D., Cawley P. (2025). T_1_ and T_2_ measurements of the neonatal brain at 7 T. Magn. Reson. Med..

[39] Verdera J.A., Bortolazzi A., Silva S.N., Payette K., Clair K.S., McElroy S., Malik S., Hajnal J.V., Tomi-Tricot R., Rutherford M.A. (2025). HERON: High-Efficiency Real-Time Motion Quantification and Re-Acquisition for Fetal Diffusion MRI. IEEE Trans. Med. Imaging.

[40] Priovoulos N., Andersen M., Dumoulin S.O., Boer V.O., van der Zwaag W. (2023). High-Resolution Motion-corrected 7.0-T MRI to Derive Morphologic Measures from the Human Cerebellum in Vivo. Radiology.

[41] Vecchiato K., Casella C., Dokumaci A.S., Siddiqui A., Egloff A., Carney O., Cleary J.O., Jarosz J., Di Ciò P., Cleri M. (2025). Ultra-High Field 7T MRI in a Drug-Resistant Pediatric Epilepsy Cohort: Image Comparison and Radiologic Outcomes. Neurology.

[42] Burkett B.J., Wong-Kisiel L.C., Chalia M., Panda A., Krecke K.N., Messina S.A., Witte R.J., Britton J.W., Brinkmann B.H., Fagan A.J. (2026). Clinical 7T MRI for Epilepsy: A Retrospective Review of 50 Cases. AJNR Am. J. Neuroradiol..

